# Endogenous sources of interbrain synchrony in duetting pianists

**DOI:** 10.1093/cercor/bhab469

**Published:** 2022-01-14

**Authors:** Katarzyna Gugnowska, Giacomo Novembre, Natalie Kohler, Arno Villringer, Peter E Keller, Daniela Sammler

**Affiliations:** Department of Neurology, Max Planck Institute for Human Cognitive and Brain Sciences, Leipzig 04103, Germany; Research Group Neurocognition of Music and Language, Max Planck Institute for Empirical Aesthetics, Frankfurt am Main 60322, Germany; Neuroscience of Perception and Action Lab, Italian Institute of Technology (IIT), Rome 00161, Italy; Department of Neurology, Max Planck Institute for Human Cognitive and Brain Sciences, Leipzig 04103, Germany; Research Group Neurocognition of Music and Language, Max Planck Institute for Empirical Aesthetics, Frankfurt am Main 60322, Germany; Department of Neurology, Max Planck Institute for Human Cognitive and Brain Sciences, Leipzig 04103, Germany; Department of Clinical Medicine, Center for Music in the Brain, Aarhus University, Aarhus 8000, Denmark; The MARCS Institute for Brain, Behaviour and Development, Western Sydney University, Sydney, NSW 2751, Australia; Department of Neurology, Max Planck Institute for Human Cognitive and Brain Sciences, Leipzig 04103, Germany; Research Group Neurocognition of Music and Language, Max Planck Institute for Empirical Aesthetics, Frankfurt am Main 60322, Germany

**Keywords:** attention, EEG hyperscanning, interactional synchrony, joint action, temporal anticipation

## Abstract

When people interact with each other, their brains synchronize. However, it remains unclear whether interbrain synchrony (IBS) is functionally relevant for social interaction or stems from exposure of individual brains to identical sensorimotor information. To disentangle these views, the current dual-EEG study investigated amplitude-based IBS in pianists jointly performing duets containing a silent pause followed by a tempo change. First, we manipulated the similarity of the anticipated tempo change and measured IBS during the pause, hence, capturing the alignment of purely endogenous, temporal plans without sound or movement. Notably, right posterior gamma IBS was higher when partners planned similar tempi, it predicted whether partners’ tempi matched after the pause, and it was modulated only in real, not in surrogate pairs. Second, we manipulated the familiarity with the partner’s actions and measured IBS during joint performance with sound. Although sensorimotor information was similar across conditions, gamma IBS was higher when partners were unfamiliar with each other’s part and had to attend more closely to the sound of the performance. These combined findings demonstrate that IBS is not merely an epiphenomenon of shared sensorimotor information but can also hinge on endogenous, cognitive processes crucial for behavioral synchrony and successful social interaction.

## Introduction

Behavioral synchrony is an important aspect of social behavior, observable in several social species (Couzin 2018; Ravignani et al. 2019). In humans, behavioral synchrony often emerges automatically (Sebanz et al. 2006; Schmidt and Richardson 2008; Marsh et al. 2009; Koban et al. 2019) and facilitates mutual affiliation, trust, and prosocial tendencies (Vicaria and Dickens 2016; Mogan et al. 2017; Gelfand et al. 2020). A multitude of cognitive and neural processes have been proposed to underpin synchrony in social interactions (Garrod and Pickering 2009; Frith and Hasson 2016; Müller et al. 2018). A related phenomenon that has attracted increasing interest is interbrain synchrony (IBS), that is, the synchronization of interactants’ neural activities, often observed during synchronous social behavior (Lindenberger et al. 2009; Dumas et al. 2010; Sänger et al. 2012; Yun et al. 2012; Müller et al. 2013; Kawasaki et al. 2018).

Over the past decade, the frequent observation of IBS across numerous social interactive tasks led to the assumption that IBS plays a functional role in social behavior (for recent reviews, see Liu et al. 2018; Mu et al. 2018; Nummenmaa et al. 2018; Wang et al. 2018; Czeszumski et al. 2020; Kingsbury and Hong 2020; Wass et al. 2020; Hoehl et al. 2021). However, which specific factors lead to IBS is currently not fully clear. On the one hand, all interactants are exposed to identical sensory input or perform the same movements during behavioral synchrony. This shared sensorimotor information is processed similarly by each individual’s brain, which may lead to spurious synchronization between brains that would also be observed in noninteractive settings (Novembre and Iannetti 2021). On the other hand, IBS may also emerge from the alignment of cognitive processes supporting social interaction, that is, mental processes that do not strictly depend on external stimuli or movements, such as joint time estimation (Mu et al. 2016, 2017; Novembre et al. 2017), or the top-down allocation of attention to important stimuli or exchanged social cues (Gvirts and Perlmutter 2020). However, it has remained unclear so far whether IBS indeed hinges on such aligned cognitive processes or can be fully explained by similar sensorimotor processes in individual brains (Lindenberger et al. 2009; Burgess 2013; Kingsbury and Hong 2020; Hamilton 2021; Novembre and Iannetti 2021). To the extent that the latter is true, IBS can be considered to be epiphenomenal in nature and unlikely to have functional significance for behavioral synchrony.

This study aims to isolate cognitive from sensorimotor drivers of IBS during behavioral synchrony. We do so by manipulating cognitive variables relevant for musical interactions in piano duos, while 1) keeping sensory input and movements as similar as possible across conditions and 2), more radically, by completely removing sensory input and movements for part of the task. Both these steps were taken to control for IBS related to a shared sensory input. Joint music making provides an excellent testbed for putting these manipulations into action (D’Ausilio et al. 2015). First, group musical performance is a natural interaction that allows targeted manipulations of cognitive aspects of social interactions, for example, who is leading or following (Vanzella et al. 2019) or how much attention musicians pay to their own or their partner’s performance (Keller 2008; Keller et al. 2014), while keeping movements and the resulting auditory outcome similar across performances. Second, although most social interactions require the exchange of sensory information, music contains natural instances in which interaction occurs with little-to-no sensory feedback and movement: during musical pauses. As a famous saying states: “Music is not in the notes, but in the silence between them.” Pauses require musicians to internally keep track of the musical tempo in order to plan the timing of their next entry as accurately and synchronously as possible, carrying on their social interaction despite the absence of direct auditory feedback or overt movements. Importantly, the regular temporal structure of music allows the similarity of co-performers’ temporal planning during the pause to be precisely controlled and manipulated by setting a specific tempo via instructions prior to performance.

**Figure 1:**
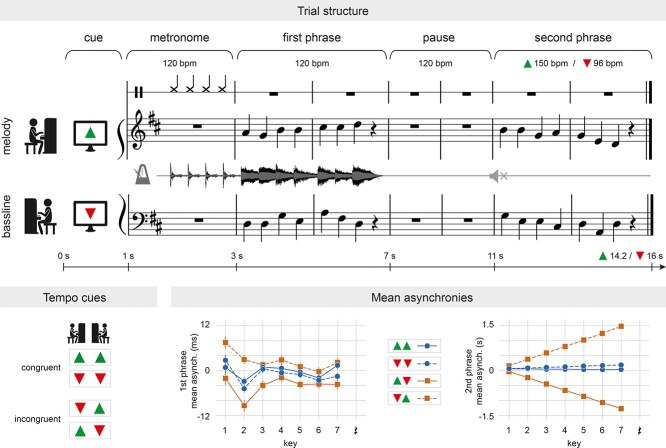
Experimental design. The upper panel illustrates the experimental setup and the time course of one example trial. The left lower panel shows the four tempo instructions (tempo cues), which result in two TEMPO conditions: speed up/speed up, slow down/slow down (both congruent) and speed up/slow down, slow down/speed up (both incongruent). The right lower panel depicts the mean asynchronies between the players’ keystrokes in the four tempo instructions in the first phrase (left) and the second phrase (right). Congruent instructions resulted in mean signed asynchronies close to zero, while up-down instructions led to negative asynchronies and down-up instructions to positive asynchronies (see main text for details). This effect was clearly observable in the second phrase, but also occurred more subtly in the first phrase (note the different time scales of the plots).

Here, we analyzed IBS in pairs of pianists in duos, as they performed complementary parts of Bach chorales (i.e., melody and bassline) that were edited to contain a silent pause. We capitalized on the dual-EEG dataset of Novembre et al. (2016) who had looked at *intra*brain neural correlates of behavioral synchrony (alpha power) during joint performance, leaving room for investigating *inter*brain dynamics during joint performance and the pause. In the paradigm of Novembre et al. (2016), two relevant factors were manipulated. First, during the 4-s pauses embedded in the chorales, the two pianists covertly planned to resume playing at a predefined faster or slower tempo (Fig. 1). Notably, unbeknownst to the pianists, the tempi they planned were either congruent (i.e., both pianists planned to speed up or to slow down) or incongruent (i.e., one pianist planned to speed up, the other to slow down and vice versa; TEMPO manipulation). This permitted us to test the dynamics of IBS in the total absence of shared sensory feedback or movements by manipulating the similarity of a purely endogenous, cognitive process—temporal planning—during the pause. To the extent that IBS does not merely depend on shared sensory information, we hypothesized that IBS should be stronger during the planning of congruent as opposed to incongruent tempi.

Second, the two pianists had or had not previously practiced the musical part performed by the other (FAMILIARITY manipulation). Motor familiarity with the partner’s part has been found to modulate the degree to which pianists integrate the sounds produced by their partners into their own action plans (Ragert et al. 2013; Novembre et al. 2016). More precisely, when pianists were unfamiliar with the other’s part, they showed a stronger (Novembre et al. 2016) and more stable mutual adaptation (Ragert et al. 2013) than during familiar pieces. This pattern of results has been proposed to emerge from a differential use of internal and external sources of information during joint performance, that is, a flexibly balanced focus on own motor knowledge (in familiar pieces) versus partner-produced sounds (in unfamiliar pieces). Attention allocation to sensory cues of coperformers’ actions has been identified as a critical cognitive factor facilitating interpersonal coordination (e.g., Temprado and Laurent 2004; Richardson et al. 2007). Moreover, joint attention to external sensory information has been associated with increased IBS, for example, between conversation partners in a multispeaker environment (Dai et al. 2018) and between partners in a joint visual search task (e.g., Koike et al. 2016; Szymanski et al. 2017). Hence, we explored the influence of the FAMILIARITY manipulation on the dynamics of IBS during joint performance with sound, assuming that it indirectly modulates the degree of mutual adaptation and joint attention to the sounds of each other’s performance. We predicted that IBS would be higher in unfamiliar than familiar pieces, although the overall movements and shared auditory outcome (two-voiced Bach chorales) remained comparable. This would provide further evidence that IBS is not slavishly driven by shared sensorimotor information but can be modulated by cognitive parameters of the interaction.

Given that the tempi used in the present experimental task were relatively slow (~1 to 3 Hz), we reasoned that a similarly slow neural signal may be most appropriate for capturing the alignment of cognitive processes underlying the planning, attention to, and performance of these tempi. We considered amplitude envelopes of the neural signal as a good candidate for this (Zamm et al. 2018, 2021), as they are thought to reflect activity fluctuations at the rate of temporally regular events: A growing number of studies have demonstrated periodic modulations of, for example, beta or gamma power (Zanto et al. 2005, 2006; Fujioka et al. 2009, 2012) or of the broadband neural signal (cf. Nozaradan 2014) at the tempo of rhythmic stimulation. Importantly, these activity modulations have been observed not only in response to regular beats or music but also during beats that were expected but not presented (Zanto et al. 2005, 2006; Fujioka et al. 2009). Moreover, peaks of neural activity at low frequencies between 1 and 5 Hz have been related not only to the tempo of an external beat (e.g., Nozaradan et al. 2015) but also to internal beat induction (Nozaradan et al. 2016) and metrical representations in the absence of explicit physical cues in the sensory signal (Nozaradan et al. 2011). Together, these data suggest that periodic amplitude modulations at the frequency of the beat capture the temporal structure of external sensory events as well as cognitive aspects of time processing including temporal prediction. This makes them a suitable tool for revealing the alignment of endogenous processes supporting joint music performance and planning.

We thus calculated how well amplitude envelopes were aligned between interacting pianists by computing the phase locking value (PLV) between the envelopes of the delta (1–3 Hz), theta (4–7 Hz), alpha (8–12 Hz), beta (13–30 Hz), and gamma band (30–40 Hz). We chose to focus on these five bands because all of them have been reported in the literature, including fronto-central delta-theta IBS (Lindenberger et al. 2009; Sänger et al. 2012; Müller et al. 2013; Müller and Lindenberger 2019, 2021), frontal and right parietal theta and beta IBS (Yun et al. 2012), bilateral posterior IBS in the alpha band (Kawasaki et al. 2018), and central and right posterior IBS in the alpha mu, beta, and gamma band (Dumas et al. 2010). To account for the diversity of reported topographies that often differ between participants and are likely to emerge from multiple sources coactivated during such a complex task, we first compared IBS globally, that is, across all electrodes. Additionally, we looked more closely at a local region of interest (ROI) over right posterior scalp regions. This ROI was chosen following the repeated observation of centro-parietal effects, often with slight right-hemispheric predominance in both *inter*brain (Dumas et al. 2010; Yun et al. 2012; Kawasaki et al. 2018) and *intra*brain analyses (Tognoli et al. 2007; Naeem, McGinnity, et al. 2012a; Naeem, Prasad, et al. 2012b) of neural correlates of behavioral synchrony, including our previous analysis of the current dataset (Novembre et al. 2016). Moreover, right posterior brain regions have been associated with the integration of self and other related information (Yang et al. 2015; Dumas et al. 2020), mutual adaptation as a synchronization strategy (Heggli et al. 2021), auditory attention (Zatorre et al. 1999; Snyder et al. 2006; Hirnstein et al. 2013), and time estimation processes crucial for establishing behavioral synchrony during interaction (Morillon and Baillet 2017).

## Methods

### Participants

Twenty-eight amateur pianists (19–33 years old, mean age ± SD: 25.36 ± 3.70 years, 9 males, 1 left-handed) with 5–21 years of musical training (mean ± SD: 13.43 ± 4.66 years) participated in the experiment. Data from three more pairs were acquired but excluded because they did not follow the instructions correctly (*N* = 2) or due to technical problems (*N* = 1). Participants were randomly paired into 14 dyads and did not know each other before the experiment. None of the participants reported a history of neurological or psychiatric disorders. They all had normal or corrected to normal vision and reported normal hearing. Participants provided written informed consent prior to the experiment and were paid for their participation. The study was approved by the local ethics committee of the University of Leipzig and conformed to the principles of the Declaration of Helsinki.

### Materials and Pre-experimental Training

Four simple, bimanual musical pieces served as material in this experiment. They were created based on Bach chorales (cf. Novembre et al. 2012, 2014, 2016) and consisted of a melody and a bassline. One pianist was required to play the melody with the right hand, while the other played the bassline with the left hand (assignment of which pianist played the melody or bassline was counterbalanced across pieces). Each piece was composed of two phrases, which contained two bars with seven crotchets and a crotchet rest, in both melody and bassline. Phrases were separated by a pause of two bars resulting in 6-bar-long pieces (see Fig. 1). Note that the crotchet rest prior to the pause served as a buffer preventing contamination of the pause by auditory or motor activity. The task required the first phrase and the pause to be performed at a tempo of 120 beats per minute (bpm), while the second phrase had to be played at a tempo of 150 bpm (faster) or 96 bpm (slower).

Participants were provided with musical scores of the pieces to practice 1 week prior to the experiment. Crucially, for two pieces, they were given scores for both the melody and the bassline, while for the remaining two pieces they received only the melody (one piece) or only the bassline (one piece) leaving participants unfamiliar with the partner’s part. The assignment of the pieces was fully counterbalanced across pairs: seven out of fourteen pairs learned pieces A and B bimanually (i.e., melody and bassline), and pieces C and D unimanually (i.e., only melody or only bassline). The remaining seven pairs learned the pieces in the opposite configuration. In addition to the scores, pianists received audio recordings of a metronome beating at 120 bpm and changing to 96 or 150 bpm to help them learn the correct tempo change in the second phrase.

### Procedure

The experimental session started with a short training procedure to make sure that pianists were able to perform the pieces fluently, that is, without pitch or rhythm mistakes and with correctly executed tempo changes. Participants were seated in two separate, acoustically shielded rooms, blocking any other channel of communication besides the digital sound of the pianos. Each room was equipped with a digital MIDI piano (Yamaha Clavinova CLP150), one pair of headphones (Sennheiser HD 280 Professional), and an EEG amplifier. A computer monitor placed on top of each piano served to display visual cues. Pianists’ behavior was monitored and recorded via video cameras (with aerial view) transmitting videos to the experimenters in the controlroom.

Each trial started with a fixation cross (duration = 500 ms) followed by a visual cue (1000 ms) indicating whether the tempo of the second phrase should be faster (green triangle pointing upward) or slower (red triangle pointing downward). Pianists saw only their own cue and were naïve about the tempo instruction given to their partner. Crucially, unbeknownst to each other, pianists were instructed to change tempo in the same (congruent) or different (incongruent) directions. That is, either both pianists speeded up or slowed down, or one pianist speeded up and the other slowed down. After the cue, participants heard four metronome beats at 120 bpm (1 bar) indicating the tempo of the first phrase and the pause, which was the same in all trials. Just after the metronome, players executed the first phrase (2 bars and 120 bpm), silently planned the tempo change in the pause (2 bars and 120 bpm), and then performed the second phrase in the new tempi (2 bars and 150/96 bpm). Pianists could hear the sound of both pianos in the first phrase, while the audio output was fully muted in the second phrase to keep them unaware of the congruent and incongruent tempo changes. Throughout the whole trial, participants were presented with the scores of only their own part, that is, either melody or bassline. The scores disappeared when the execution of the second phrase was supposed to be completed according to the new tempo. Stimulus presentation, registration of piano key presses, regulation of audio-feedback, and EEG triggering for both pianists were controlled by one computer with Presentation software (Version 14.9, Neurobehavioral Systems, Inc.; for more technical details, see Novembre et al. 2016).

Each pair completed 48 trials in each of the four conditions (according to the 2 × 2 factorial design crossing congruent and incongruent TEMPO changes and FAMILIARITY with the partner’s part) resulting in 192 trials in total. The experiment lasted around 60 min, interrupted by a 10-min break after half of the trials.

### E‌EG Data Acquisition

EEG was recorded from 29 Ag/AgCl electrodes (FP1, FPZ, FP2, F7, F3, FZ, F4, F8, FC5, FC1, FC2, FC6, T7, C3, CZ, C4, T8, CP5, CP1, CP2, CP6, P7, P3, PZ, P4, P8, O1, OZ, and O2) arranged in elastic caps (Electro-Cap, MES Forschungssysteme GmbH, Munich, Germany) according to the extended 10–20 system (Lagerlund et al. 1993). An electrode placed on the left mastoid served as reference during recording; an additional electrode was placed on the right mastoid for later offline re-referencing. The ground electrode was placed on the sternum. Horizontal and vertical electrooculograms (EOG) were recorded from electrodes placed on the outer canthus of both eyes and above and below the right eye. Impedances were kept below 5 kΩ. During recording, EEG signals were amplified by two separate 72-channel Refa amplifiers (24-bit, Twente Medical Systems International, Oldenzaal, The Netherlands) at a sampling rate of 500 Hz and passed through an anti-aliasing filter with a cutoff at 0.27*sampling rate, that is, 135 Hz. Recording was managed by in-house software Qrefa on two computers with identical hardware that both received EEG triggers from the computer controlling stimulus presentation and behavioral response registration (see above).

### Behavioral Data Analysis

#### Behavioral Data Cleaning

Behavioral data were analyzed similarly to Novembre et al. (2016). Only trials without key errors and correct tempo changes were analyzed (for more details, see Novembre et al. 2016). In total, 86.4% of the trials were included to calculate measures of synchronization accuracy and mutual adaptation between participants.

#### Calculation of Synchronization Accuracy and Mutual Adaptation

Synchronization accuracy was operationalized as mean absolute keystroke asynchronies between participants (Keller et al. 2007; Ragert et al. 2013). Like Novembre et al. (2016), we calculated the time differences (asynchronies) between complementary keystrokes (i.e., keystrokes that were meant to be played synchronously according to the musical score). We then normalized these values to account for the metric position of each key and the slightly variable spatial distance between keys within each piece (Wing et al. 2014). Normalization was done within each pair by subtracting (trial-by-trial) the average signed asynchrony separately for each keystroke position and piece. These asynchronies were then averaged across trials for each of the four tempo instructions (up-up, down-down, up-down, and down-up), converted into absolute values, and then averaged across pieces according to the 2 × 2 factorial design. To assist the interpretability of the IBS results, we quantified synchronization accuracy separately for the beginning and the end of the first phrase by averaging asynchronies associated with the first (early) or last (late) three keystroke positions (the fourth keystroke was omitted). This refines the analysis of Novembre et al. (2016), who focused on the first phrase as a whole.

The strength of mutual adaptation during the first phrase was estimated by cross-correlating players’ inter-keystroke intervals (IKI) and extracting the zero-lag coefficient. This value is inversely related to mutual adaptation, that is, more negative coefficients are suggestive of stronger adaptation (e.g., Repp and Keller 2008; Konvalinka et al. 2010; Konvalinka and Roepstorff 2012). Other lags (e.g., lag +1 or lag −1) informing about directional adaptation (i.e., how strongly partners were leading or following) were not analyzed because the present study did not manipulate leader-follower roles and was designed to keep roles as balanced as possible (e.g., by cueing the performance onset by the metronome and not by either of the participants and by counterbalancing left- and right-hand performance across trials; see above). The lag-0 coefficients were Fisher-z transformed (to convert their distribution from uniform to normal) and averaged according to the 2 × 2 factorial design.

Finally, as behavioral measures of joint planning outcome, we estimated 1) how synchronously the pianists started playing after the pause and 2) how similar pianists’ tempi were during the second phrase. This was done by computing 1) the mean absolute asynchronies of the first keystroke of the second musical phrase and 2) the mean absolute differences between the IKIs of the two players in the second phrase.

#### Statistical Analysis

Mean absolute asynchronies of the first phrase were statistically evaluated with a 2 × 2 × 2 repeated measures ANOVA (rmANOVA) with factors TEMPO (congruent vs. incongruent), FAMILIARITY (familiar vs. unfamiliar), and PHRASE HALF (first half vs. second half). Lag-0 cross-correlation coefficients of the first phrase, mean absolute asynchronies of the first keystroke after the pause, as well as mean absolute IKI differences of the second phrase were compared in separate 2 × 2 rmANOVAs with factors TEMPO and FAMILIARITY. All analyses were programmed in R (version 4.0.2, R Core Team 2020).

### E‌EG Data Analysis

#### E‌EG Data Preprocessing

EEG data were analyzed using FieldTrip (Oostenveld et al. 2011; downloaded on 22 June 2018) and custom MATLAB scripts (version 9.7.0.1190202, R2019b). Raw data were preprocessed as follows: we first bandpass filtered the data between 0.5 and 95 Hz and applied a notch filter between 45 and 55 Hz to eliminate line noise (both two-pass Butterworth filters, third order). Data were then re-referenced to linked mastoids and epoched between −1 and 16 s relative to the onset of the visual cue, covering the presentation time of the visual cue (1 s), the metronome (2 s), the first phrase (4 s), the pause (4 s), and the second phrase (3.2 or 5 s in fast and slow trials, respectively, see Fig. 1). Stereotypical artifacts (i.e., blinks, saccades, and muscle activity) were corrected using independent component analysis (ICA). Trials with remaining artifacts were manually rejected. Only trials with clean EEG data (and correct behavioral performance) in both pianists were analyzed further. In total, 76% of the trials were retained, that is, an average of 145.57 ± 16.90 (SD) trials per pair and 36 ± 1.60 (SD) for each of the four conditions.

#### Calculation of IBS—Phase Locking of Amplitude Envelopes

**Figure 2:**
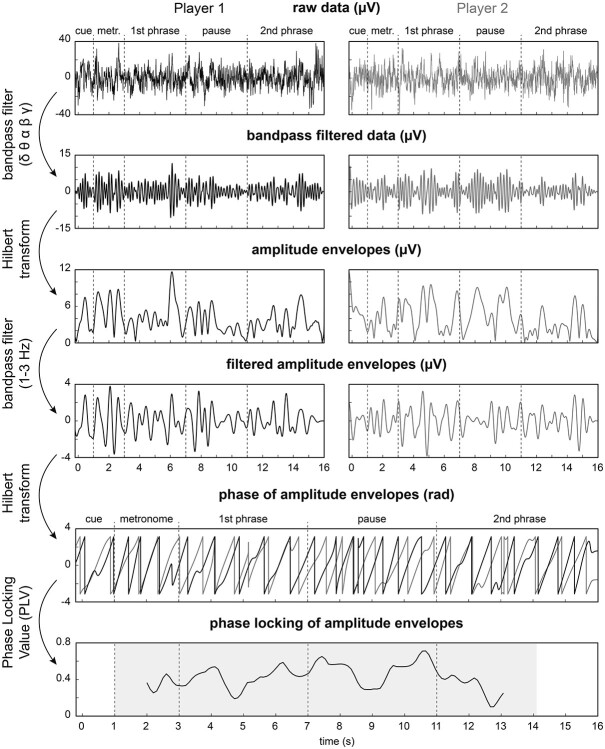
Calculation of amplitude-based IBS. The flowchart summarizes the transformation steps from raw dual-EEG data to the phase locking of amplitude envelopes in one trial for the Cz electrodes of two pianists. The shaded area (bottom panel) indicates all the data covered by the sliding time windows during PLV calculation (see Methods).

**Figure 3:**
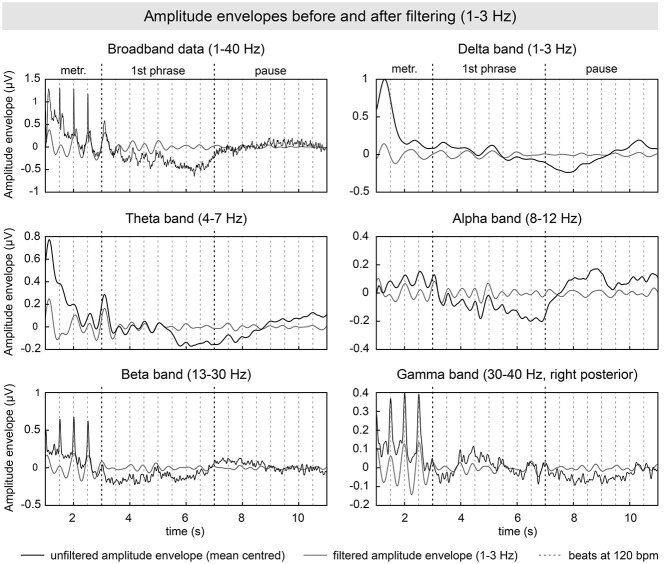
Amplitude envelopes of the EEG delta, theta, alpha, beta, gamma band, and broadband data averaged across all electrodes (except for gamma, which focused on the right-posterior ROI), all participants, and all trials. Envelopes are shown before (black solid line) and after application of the 1–3 Hz bandpass filter (gray solid line), chosen to increase SNR at the tempi of the task (96, 120, and 150 bpm = 1.6, 2, 2.5 Hz). Dashed vertical lines indicate the beat timing at 120 bpm. The first beat of the first phrase and the pause are depicted in black. Note that envelopes peaked on every beat (broadband, theta, alpha, beta, and gamma bands) or every second beat of the task (delta band). Unfiltered amplitude envelopes were mean centered per trial to be visually comparable with filtered amplitude envelopes.

We calculated the phase locking of amplitude envelopes of the neural signal as measure for IBS (see Fig. 2 for the workflow). Therefore, we first bandpass filtered the data in five frequency bands (delta: 1–3 Hz, theta: 4–7 Hz, alpha: 8–12 Hz, beta: 13–30 Hz, and gamma: 30–40 Hz; two-pass Butterworth filter, fourth order) and applied a Hilbert transform to extract the amplitude envelope. As can be seen in Figure 3 (black solid lines), these envelopes showed periodic modulations related to the temporal regularity of the paradigm, that is, modulations peaking on every beat in the theta, alpha, beta, and gamma bands and on every second beat in the delta band, both in the metronome/first phrase as well as in the pause (although weaker). In order to increase the signal-to-noise ratio (SNR) of these modulations, we bandpass filtered the envelopes in the frequency range of pianists’ planned and performed musical tempi (1–3 Hz, two-pass Butterworth, fourth order; see gray solid lines in Fig. 3). Note that the 1–3 Hz range encompasses both the slower (96 bpm = 1.6 Hz) and the faster (150 bpm = 2.5 Hz) musical tempi of the second phrase, as well as the tempo of the metronome, first phrase and pause (120 bpm = 2 Hz).

We then extracted the phase of these envelopes by applying a second Hilbert transform, and finally calculated the PLV between homologous electrodes of the two players (i.e., C3-C3, C4-C4, etc.). We focused on homologous electrode pairings because we were interested in the alignment of analogous cognitive processes, that is, processes that are likely to show a similar scalp topography in both pianists at the same time. Furthermore, this approach allowed us to minimize the number of multiple comparisons. PLVs were calculated using a moving time window (width = 2 s, moving in steps of 100 ms). The first time window started at the onset of the metronome (i.e., 1 s after trial onset) and the last time window ended where trials with fast tempo (150 bpm) were supposed to be completed (i.e., 14.2 s after trial onset, which was the last time point for which data for both participants were available across conditions; see gray shading in the lowest panel of Fig. 2). PLVs were calculated for each time window *w* per trial *n* with the following formula (adapted from Lachaux et al. 1999; Cohen 2014):

$$ {\mathrm{PLV}}_{\left(w,n\right)}=\frac{1}{T}\left|\sum_{t=1}^T{e}^i\left({\theta}_{1\left(t,w,n\right)}-{\theta}_{2\left(t,w,n\right)}\right)\right| $$

where $({\theta}_{1(t,w,n)}-{\theta}_{2(t,w,n)})$ stands for the phase difference between the amplitude envelopes of the two brains at time point *t* in time window *w* in trial *n*; *e* stands for Euler’s number and *i* for imaginary number. The obtained PLVs were then averaged within ROIs (across all or right-posterior electrodes) and subsequently across trials per condition within pairs.

#### Statistical Analysis and Correction for Multiple Comparisons

In order to reduce the dimensionality of the data, we performed the analysis in two modes: global and local. For the global analysis, we averaged PLVs across all electrodes, further referred to as global region of interest (GLOBAL ROI). For the local analysis, PLVs were averaged across electrodes C4, CP2, CP6, P4, and P8 forming a right-posterior region of interest (RP ROI). As mentioned in the Introduction, the GLOBAL ROI was chosen to account for the heterogeneity and individual variability of topographies reported in the literature, likely to stem from multiple coactivated sources. The RP ROI was chosen following the repeated observation of right centro-parietal effects in dual interactive setups (Tognoli et al. 2007; Dumas et al. 2010; Naeem, McGinnity, et al. 2012a; Naeem, Prasad, et al. 2012b; Kinreich et al. 2017; Levy et al. 2017; see Gvirts and Perlmutter 2020 for an fNIRS hyperscanning review). Averaged PLVs in each ROI were then entered into 2 × 2 rmANOVAs with the factors TEMPO and FAMILIARITY. ANOVAs were calculated separately for each time point and frequency band, resulting in time series of *F*-values (see Figs 5 and 6). Cluster-based permutation tests were used to control for multiple comparisons across time points and frequency bands (Maris and Oostenveld 2007). Following the approach of Novembre et al. (2019) and Pan et al. (2021), we first identified clusters with at least two consecutive time points with *P*-values < 0.01 (to control for five frequency bands), and summed the *F-*values in each cluster. Next, we permuted the assignment of trials to the experimental conditions (number of permutations *N* = 1000), each time saving the sum of *F*-values obtained for the biggest clusters in these random datasets. This way we obtained a random distribution of cluster *F*-values. This distribution was used to define a cluster’s significance threshold (*P* = 0.05) against which the significance of the real clusters was assessed.

### Brain–Behavior Relationship

To explore the relationship between IBS and behavioral synchrony, we ran four linear mixed models on single trial data, using the function *lmer* of the R package lme4 version 1.1-27 (Bates et al. 2015). Averaged PLVs within significant time windows (see Results) were used as predictors for corresponding behavioral measures (see Results). Participant pair was specified as random intercept in all models. All models followed the general form: lmer (behavior ~ PLV + (1|pair)). A total of 2038 trials from 14 pairs entered the analyses. Raw behavioral data were used in order to retain the original trial-wise relationship between the neural and behavioral measures and to avoid biasing the estimation of the random structure. Assumptions for linear mixed models (e.g., normal distribution of residuals, homogeneity of residuals, and model stability) were tested and met. If necessary, skewed data were log- or square-root-transformed to obtain normal distribution. The only exceptions were the mean absolute IKI differences in the second phrase that showed a bimodal distribution reflecting congruent and incongruent tempi between partners. These IKI differences were binned into two categories by median split and entered into a logistic generalized mixed model. Logistic mixed models do not require normal distribution of residuals and are, hence, well suited for predicting binary responses. Significance of all models was assessed by comparison with a null model that preserved the random structure without fixed effects.

### Control Analyses

#### Relationship between PLVs and Underlying Power

PLVs can be influenced by the underlying power of the signals used to compute the PLV (Van Diepen and Mazaheri 2018). To make sure that the observed PLV differences were not driven by power differences between experimental conditions, we averaged power (extracted using a bandpass filter and a Hilbert transform) in the respective ROIs, frequency bands, time windows, and conditions in which we observed significant PLV differences. These values were then compared across conditions by means of paired-samples *t*-tests.

#### Baseline PLV

The magnitude of a PLV is difficult to interpret because it not only depends on the experimental manipulation but also on other factors such as the temporal structure common to all trials (i.e., the shared tempo of 120 bpm in the first phrase and the pause). To gain a “baseline” PLV driven by this common temporal structure of action independently of experimental manipulation, we randomly paired trials within each dyad and computed the average PLV as above. Real PLVs and baseline PLVs were compared by means of paired-samples *t*-tests. This analysis served to explore the direction of the effects observed in the main analyses, that is, whether a specific manipulation led to a genuine increase or decrease of IBS.

#### Surrogate PLV

To test for effects attributable to social interaction, we generated a surrogate dataset by randomly pairing players who had not actually performed with each other, while matching trials belonging to the same experimental condition. Trial numbers were equalized between players by either deleting spare trials or duplicating the first few trials in player 2 to match the trial numbers of player 1. This way, the analyses of real and surrogate data had exactly the same statistical power. PLVs of surrogate pairs were used to assess whether synchronization was specific to the social interaction within real pairs or could be explained by the similarity of individual actions dictated by our experimental setup. Data underwent the same cluster-based permutation test as PLVs of real pairs.

## Results

### Behavioral Results

#### Synchronization Accuracy in the First Phrase

**Figure 4:**
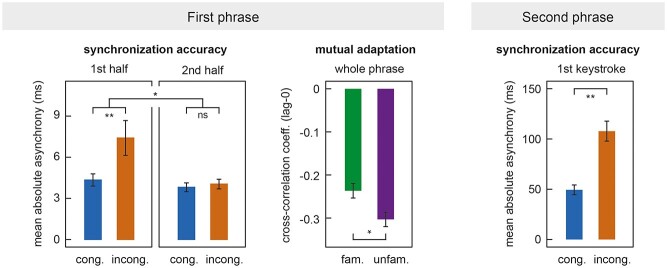
Behavioral results. Left panel: Synchronization accuracy between players in the first phrase. Higher values denote lower synchronization accuracy. First half refers to keystrokes 1–3 and second half to keystrokes 5–7 in the first phrase. Middle panel: Mutual adaptation of players in the first phrase. Lower values denote stronger mutual adaptation. Right panel: Synchronization accuracy between players at the first keystroke after the pause. Cong., congruent; incong., incongruent; fam., familiar; unfam., unfamiliar. Error bars indicate ±1SEM.

The rmANOVA with factors TEMPO (congruent/incongruent), FAMILIARITY (familiar/unfamiliar), and PHRASE HALF (first half/second half) on mean absolute asynchronies in the first phrase of the musical pieces showed that participants were behaviorally more synchronized in congruent than in incongruent trials (main effect of TEMPO: *F*(1,13) = 11.92, *P* = 0.004, ${\eta}_P^2$ = 0.48). A significant interaction of TEMPO × PHRASE HALF (*F*(1,13) = 5.26, *P* = 0.039, ${\eta}_P^2$ = 0.29) showed that this difference was particularly pronounced at the beginning of the phrase, whereas players achieved tight synchrony in all conditions in the second half of the phrase (see left panel in Fig. 4). Post hoc comparisons confirmed significantly higher asynchronies in incongruent than congruent trials in the first half (*t*(13) = −3.22, *P* = 0.007), but not in the second half of the first phrase (*t*(13) = −0.42, *P* = 0.683), refining the results of Novembre et al. (2016). No effect of FAMILIARITY was found (*F* < 1).

#### Mutual Adaptation in the First Phrase

Analysis of lag-0 cross-correlations revealed that pianists were adapting more strongly to each other in unfamiliar than familiar pieces, as shown by significantly more negative lag-0 cross-correlations (main effect of FAMILIARITY: *F*(1,13) = 5.05, *P* = 0.043, ${\eta}_P^2$= 0.28; see middle panel in Fig. 4). No main effect of TEMPO (*F* < 1) or TEMPO × FAMILIARITY interaction (*F* < 1) was found (see also Novembre et al. 2016).

#### Synchronization Accuracy at the Onset of the Second Phrase

The first keystroke after the pause was produced significantly more synchronously, that is, with smaller mean absolute asynchrony, in the congruent compared to the incongruent conditions (main effect of TEMPO: *F*(1,13) = 9.46, *P* = 0.009, ${\eta}_P^2$= 0.42; see right panel in Fig. 4). Note that the two players should have produced this keystroke similarly synchronously irrespective of the TEMPO condition, because the pause always included eight beats at 120 bpm (i.e., a duration of 4 s). We ascribe the observed asynchronies to differences in temporal planning, that is, the anticipation of different new tempi after the pause. No main effect of FAMILIARITY (*F* < 1) or TEMPO × FAMILIARITY interaction (*F* < 1) was found.

#### Execution of the New Tempo in the Second Phrase

Mean absolute IKI differences in the second phrase were significantly smaller in trials with congruent (*M ±* SEM: 54.28 ± 1.20 ms) than with incongruent tempo changes (*M ±* SEM: 215.29 ± 1.83; main effect of TEMPO: *F*(1,13) = 425.95, *P* < 0.001, ${\eta}_P^2$= 0.97) showing that pianists changed tempo in the second phrase as instructed. No main effect of FAMILIARITY or TEMPO × FAMILIARITY interaction was found (*F*s < 1).

### E‌EG Results

#### IBS during the First Phrase

##### Familiarity Effect

**Figure 5:**
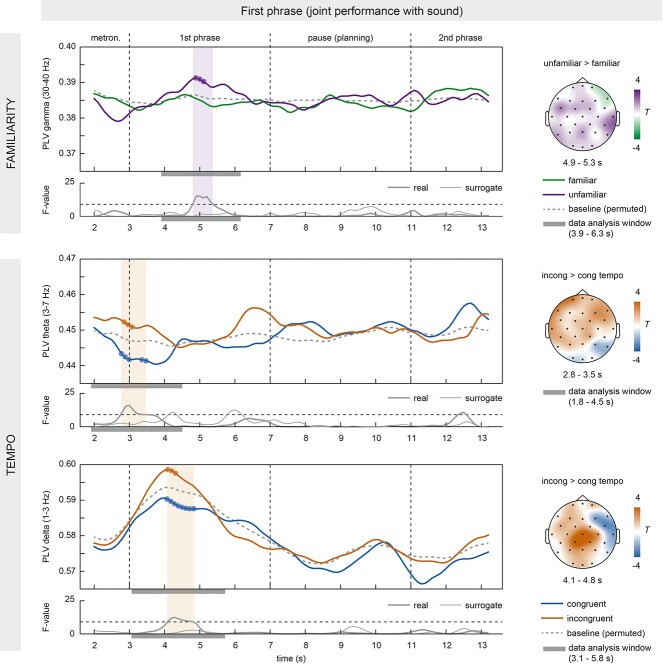
Stronger IBS during unfamiliar pieces and incongruent tempi in the first phrase. PLV time courses in the gamma band (30–40 Hz) showed higher IBS in unfamiliar (purple) than familiar pieces (green) across all electrodes. Moreover, IBS in theta (3–7 Hz) and delta bands (1–3 Hz) was higher at the beginning of trials with incongruent (red) compared to congruent tempo instructions (blue) across all electrodes. Topography plots depict PLV differences in significant time windows, marked as shaded areas in the PLV time courses. The gray bars under the time courses of PLV and *F*-values indicate the data analysis window that contributed to the effect. Time courses of *F*-values illustrate significance in real pairs (thick line) but not surrogate pairs (thin line) after correction for multiple comparisons (threshold depicted as dashed line). The dashed gray line in the PLV time courses represents the baseline PLV after permuting trials within each pair. Small circles indicate significant difference from baseline PLV (*P* < 0.05, uncorrected).

In the first phrase of the musical duets, IBS was higher in unfamiliar than familiar pieces in the gamma band (30–40 Hz) across all electrodes. PLVs differed significantly between 4.9 and 5.3 s (covering data from 3.9 to 6.3 s, that is, nearly the entire first phrase; main effect of FAMILIARITY: sum of *F*s(1,13) = 70.29, *P* = 0.047, cluster-corrected; see Fig. 5). No significant effects were found in other frequency bands. Compared to baseline PLV, IBS increased in unfamiliar pieces (circles in Fig. 5). The effect could not be explained by gamma power differences between conditions (gamma power between 4.9 and 5.3 s: *t*(13) = 1.604, *P* = 0.120). Moreover, no significant clusters were found in surrogate pairs (see thin line in the time course of *F*-values in Fig. 5).

Although a similar main effect of FAMILIARITY was found in behavioral measures of mutual adaptation, a linear mixed model exploring the relationship between lag-0 cross-correlation coefficients and average gamma PLV (4.9–5.3 s in the GLOBAL ROI) did not reach significance (*P* > 0.401).

##### Tempo Effect

At the beginning of the first phrase, we found an additional, nonhypothesized effect: IBS was higher for incongruent than congruent tempo conditions in the delta and theta bands (1–3 Hz and 3–7 Hz) across all electrodes (see Fig. 5). In theta, PLVs differed significantly between 2.8 and 3.5 s (covering data from 1.8 to 4.5 s; main effect of TEMPO: sum of *F*s(1,13) = 93.88, *P* = 0.025, cluster-corrected), that is, around the onset of the joint performance, including the last two metronome beats and the first four keystrokes. Note that the effect cannot be driven by the metronome, which was identical across conditions. In delta, PLV differences occurred between 4.1 and 4.8 s (sum of *F*s(1,13) = 84.80, *P* = 0.037, cluster-corrected, covering data from 3.1 to 5.8 s), that is, in the first half of the first phrase when performance was slightly asynchronous between partners (see left panel of Fig. 4). Compared to baseline, IBS increased in incongruent and decreased in congruent trials (see small circles in Fig. 5). Effects were not explained by delta or theta power differences between conditions (theta between 2.8 and 3.5 s: *t*(13) = 0.248, *P* = 0.806; delta between 4.1 and 4.8 s: *t*(13) = 0.155, *P* = 0.878). No significant clusters were found in surrogate pairs (see thin line in the time course of *F*-values in Fig. 5).

Given that a similar main effect of TEMPO was also found in behavioral synchronization accuracy, particularly at the beginning of the first phrase, two linear mixed models were computed to explore the relationship between mean absolute asynchronies of the first three keystrokes and average delta and theta PLV (in the significant time windows in the GLOBAL ROI). None of the models reached significance (*P*s > 0.200).

#### IBS in the Pause

##### Tempo Effect

**Figure 6:**
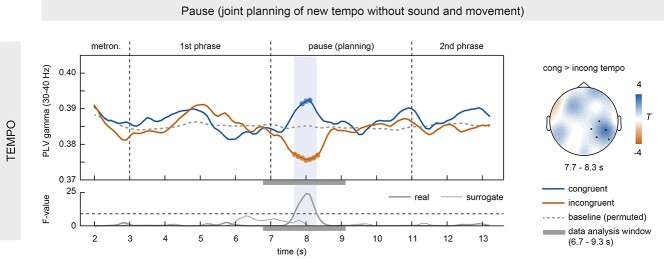
Stronger IBS during congruent tempo planning in the pause. PLV time courses in the gamma band (30–40 Hz) revealed higher IBS at right-posterior electrodes when pianists planned congruent (blue) compared to incongruent (red) tempi in the pause (see PLV difference in the topography plot). The shaded area in the PLV curves highlights the time of significant differences after correction for multiple comparisons, as can also be seen in the time course of *F*-values underneath. The thick solid line represents *F*-values for real pairs; the thin solid line refers to surrogate pairs. The gray bars under the time courses of PLV and *F*-values indicate the data analysis window that contributed to the effect. The dashed gray line in the upper panel represents the time course of baseline PLV after permuting trials within each pair. Differences between baseline PLV and congruent/incongruent conditions are marked by small circles (*P* < 0.05, uncorrected).

Pianists showed higher right-posterior IBS in the gamma band (30–40 Hz) when they planned congruent compared to incongruent tempi in the pause (main effect of TEMPO in the RP ROI: sum of *F*s(1,13) = 123.01, *P* = 0.014, cluster-corrected; see Fig. 6). The effect was significant between 7.7 and 8.3 s of the trial (calculated from 6.7 to 9.3 s of the data), that is, in the last 300 ms of the first phrase and the first half of the pause. Note that the first phrase ended on a crotchet rest, that is, a silent beat without a keystroke movement, making contamination of this effect with task-related motor activity very unlikely. No other significant clusters were found in any of the other frequency bands or ROIs. Moreover, no significant clusters were found in an exploratory analysis in a homologous left-posterior ROI including electrodes C3, CP1, CP5, P3, and P7. IBS differences (congruent minus incongruent) were nominally larger in the right (*M* ± SEM: 0.0143 ± 0.003) than in the left-posterior ROI (*M* ± SEM: 0.0049 ± 0.005) in the time window of the effect (7.7–8.3 s), although the direct comparison was not significant (paired-samples *t*-test: *t*(13) = 1.63, *P* = 0.13). Compared to baseline, IBS increased in congruent trials and decreased in incongruent trials (see small circles in Fig. 6). This effect could not be explained by gamma power differences between conditions (gamma power comparison between 7.7 and 8.3 s in the RP ROI: *t*(13) = −0.354, *P* = 0.726). No significant clusters were found in surrogate pairs (see thin line in the time course of *F*-values in Fig. 6). No effect of FAMILIARITY was found.

Higher gamma IBS in the pause was expected to be associated 1) with lower mean absolute asynchronies at the first keystroke after the pause and 2) smaller mean absolute IKI differences between participants in the second phrase. The logistic generalized mixed model showed that higher gamma IBS values indeed predicted smaller IKI differences (estimate ± SEM = −1.03 ± 0.45, *P* = 0.02, full-null model comparison: χ^2^ = 5.34, df = 1, *P* = 0.02). A similar relationship was observed between gamma IBS and mean absolute asynchronies at the first keystrokes after the pause, although it did not reach significance (estimate ± SEM = −1.94 ± 1.46, *P* = 0.18; full-null model comparison: χ^2^ = 1.78, df = 1, *P* = 0.18).

## Discussion

This study contributes to the debate whether IBS during behavioral synchrony merely reflects the similar tracking of shared sensorimotor information by each individual’s brain or can stem from aligned cognitive processes relevant for social interaction (Lindenberger et al. 2009; Burgess 2013; Kingsbury and Hong 2020; Hamilton 2021; Novembre and Iannetti 2021). To disentangle these views, we manipulated cognitive processes relevant for joint music performance while 1) keeping sensory input and movements comparable across conditions and, more notably, by 2) capitalizing on natural instances of silent joint action planning, that is, musical pauses without sensory input or movement. We calculated amplitude-based IBS, that is, the PLV between the periodically modulated amplitude envelopes extracted for each of five frequency bands (delta, theta, alpha, beta, and gamma). During joint performance with sound, we found stronger IBS in the gamma band when pianists were unfamiliar (compared to familiar) with each other’s parts. Moreover, we found stronger delta and theta IBS at the beginning of the first phrase when pianists had received incongruent tempo instructions. These effects are proposed to evolve from enhanced joint attention and mutual adaptation to the sounds of each other’s performance. Most crucially, we also found stronger IBS during musical pauses without sound or movement, in the gamma band at right-posterior electrodes, when pianists planned to change tempo in the same (compared to opposite) directions after the pause. Together, these data demonstrate that IBS does not merely depend on shared sensorimotor information but can also emerge endogenously, from aligned cognitive processes supporting social interaction. The absence of comparable IBS effects in surrogate pairs, and the predictability of future tempo relationships between partners from their IBS during the pause are further arguments for the functional relevance of IBS for social interaction, as will be discussed inturn.

### IBS during Joint Performance with Sound

During joint performance with sound in the first phrase, we observed higher IBS in the gamma band across all electrodes when pianists were unfamiliar (compared to familiar) with their partner’spart. This effect was accompanied by stronger behavioral adaptation reflected in more negative lag-0 cross-correlations (although IBS did not significantly predict behavioral adaptation on a trial-by-trial basis).

We attribute the IBS increase in unfamiliar pieces to stronger joint attention to the sounds of the performance. Note that auditory input and pianists’ movements were comparable across familiar and unfamiliar trials in terms of mean absolute asynchronies, overall tempo (120 bpm) and two-voiced content (melody and bassline), making it unlikely that IBS merely increased due to properties of shared sensorimotor information. Rather, we argue that the observed IBS modulation emerged from a different focus of pianists, on their own internal motor knowledge in familiar pieces (Keller et al. 2007; Ragert et al. 2013) or on each other’s sounds in unfamiliar pieces (see also Novembre et al. 2016). It seems plausible that lacking knowledge of the partner’s part in unfamiliar pieces made the shared sounds the only source of information that both pianists necessarily had to attend (and adapt) for synchronizing their performances (Keller et al. 2014; note that pianists did not see each other). Moreover, the stronger mutual adaptive behavior during unfamiliar pieces, as reflected in more negative lag-0 cross-correlations, suggests that pianists took other-produced sounds more into account during performance when they had not previously practiced the other’s part. This is in line with previous studies showing more stable behavioral synchrony between partners that attended to partner-produced sensory cues (Temprado and Laurent 2004; Richardson et al. 2007). Furthermore, modulations in gamma power have been previously associated with the orientation of attention toward external auditory stimuli versus internal thoughts (Villena-González et al. 2018) and the top-down allocation of auditory attention (Debener et al. 2003). Interestingly, increases in gamma power-based IBS have been observed during periods of high behavioral synchrony (Kinreich et al. 2017; Levy et al. 2017), and social attention to others’ actions has been linked to distributed frontal and temporo-parietal regions (Gvirts and Perlmutter 2020), in line with the broad distribution of our effect likely to stem from multiple sources that synchronize between partners when attention and adaption to the sensory outcomes of the other’s action is high. More generally, several EEG and fNIRS studies reported increased IBS during periods of high joint attention, for example, between adults and infants when listening to nursery rhymes (EEG theta and alpha bands, Leong et al. 2017) or children’s stories (fNIRS, Piazza et al. 2020). Together, these findings make it plausible that the observed IBS modulation emerged from differences in joint attention and adaptation to the sound of the interaction.

It could be argued that joint attention and adaptation to sounds enhance IBS by facilitating the neural tracking of this shared auditory input in each individual brain (Doelling and Poeppel 2015; Nozaradan et al. 2016; Harding et al. 2019; Lakatos et al. 2019), still not fully dissociating internal cognitive from external sensory drivers of IBS. Indeed, recent studies have shown that neural activity of individual listeners synchronizes more strongly with attended than with unattended auditory streams (Mesgarani and Chang 2012; Zion Golumbic et al. 2013; Brodbeck et al. 2018; Fiedler et al. 2019; see Obleser and Kayser 2019 for a review). Moreover, IBS was found to be higher between listeners and an attended compared to an unattended speaker (Dai et al. 2018). While these findings suggest that attention-modulated stimulus-to-brain synchronization may contribute to IBS between interaction partners (Pérez et al. 2017), it remains to be determined whether this relationship necessarily makes IBS a sensory-driven epiphenomenon or rather reflects the top-down amplification of a natural neural mechanism to facilitate social interaction. The links between IBS, attention-modulated neural tracking of shared sensory information, and their functional relevance for behavioral synchrony warrants further investigation.

### IBS during Musical Pauses without Sound

Our second manipulation tackled the challenge of shared sensorimotor information yet more rigorously by measuring IBS during instances of musical interactions without sensory input and movement, that is, musical pauses. Silent pauses of varying length are natural ingredients of music. They require musicians to precisely keep track of the musical tempo in order to resume playing in synch from the first note on after the pause. Hence, ensemble musicians pausing in between two phrases are not simply taking a break: they are actively planning their joint entry and the tempo of future performance in the absence of direct sensory feedback (Bishop and Goebl 2015). We observed increased IBS in the gamma band at right-posterior electrodes when pianists were planning to change tempo into the same compared to opposite directions, that is, when both were planning to speed up or to slow down after the pause. Moreover, IBS in the pause significantly predicted future tempo relationships between partners in the second phrase.

We attribute these modulations of IBS to the alignment of internal timekeepers in co-performers’ brains (i.e., sensorimotor neural activity supporting rhythm perception and production as discussed, e.g., by Morillon and Schroeder 2015; Paton and Buonomano 2018; Rimmele et al. 2018). Posterior gamma power is a plausible marker of internal timekeeping and temporal planning without sensory cues. For instance, the combined frameworks of active sensing and dynamic attending posit that gamma amplitude modulations, as found here, underpin temporal expectations in the absence of rhythmic cues (Schroeder and Lakatos 2009; Henry and Herrmann 2014). Accordingly, induced gamma power in auditory cortices has been found to reliably peak at the anticipated time of a beat in trains of regularly timed sounds, even when the beat was not presented (Snyder and Large 2005; Zanto et al. 2005, 2006; Fujioka et al. 2009). Furthermore, the power envelope of high gamma in auditory cortices has been shown to faithfully track the timing (and other properties) of the sound envelope of imagined musical pieces (Martin et al. 2018; Ding et al. 2019). Hence, IBS in the gamma band may reflect the interpersonal alignment of estimated time points characterized by modulations in gamma power, that is, the alignment of pianists’ internal timekeepers.

Importantly, it has been shown that internal timekeepers are biased toward new, anticipated tempi already ahead of time, reflected in subtle anticipatory tempo changes (in the range of milliseconds) well before the actual tempo change is executed (Repp 2001; Repp and Keller 2004). Indeed, although our pianists were supposed to maintain a joint tempo of 120 bpm both during the first phrase and during the pause, we found evidence for slight deviations in tempo. This is most clearly evident at the beginning of the first phrase where mean absolute keystroke asynchronies between pianists were increased when they had received opposite tempo instructions for the second phrase (i.e., one pianist to speed up, the other to slow down, and vice versa), although pianists rapidly establish synchrony upon detection of these asynchronies (see left panel in Fig. 4 and below for further discussion). We propose that similar subtle shifts in internal timekeeping reemerge when sound is no longer available, accounting for the modulations of gamma IBS during the pause. The planning of congruent tempo changes would bias timekeepers (and modulations of gamma activity) into the same direction, resulting in enhanced IBS compared to baseline, while the anticipation of incongruent tempo changes would induce misalignments between timekeepers, resulting in reduced IBS compared to baseline (see Fig. 6).

It might be argued that similar effects would also be found in two individuals anticipating congruent or incongruent prelearned tempo changes independently from each other. However, the results of our surrogate pair analysis speak against this interpretation: gamma IBS differed between congruent and incongruent anticipated tempi only in real pairs, not in surrogate pairs. This indicates that temporal planning and gamma IBS in real pairs may have been additionally influenced by the temporal fine-structure specific to the interaction of a particular dyad. In fact, each performance and trial differs slightly in timing and each pianist has an individual temporal fingerprint (e.g., due to personal artistic expression or neuromuscular differences; Keller et al. 2007; Van Vugt et al. 2013; Zamm et al. 2016), both of which have to be taken into account by interaction partners when keeping the tempo and planning their joint entries during the pause. Indeed, recent studies consistently found increased interbrain and behavioral synchrony in dyads that had interacted together before (Koike et al. 2016; Nozawa et al. 2019). Similarly, behavioral studies on piano duos showed that temporal anticipation becomes partner-specific over the course of the interaction (Ragert et al. 2013). Based on these combined findings, we argue that the observed IBS modulations emerged in real but not surrogate pairs because pianists integrated their partner’s timing into their own temporal plans. This idea might find further support in the right-posterior topography of our effect, compatible with activity in the right temporo-parietal junction (TPJ) and posterior superior temporal sulcus (pSTS). Both areas are known as hubs involved in the processing of self- and other-related information (Yang et al. 2015; Dumas et al. 2020), and self-other integration (Fairhurst et al. 2013; Heggli et al. 2021). The source localization of the observed IBS effect in these regions can be clarified in future studies with high-density EEG recordings. Overall, the data provide strong evidence that other-related information can influence own internal temporal plans and modulate IBS despite the absence of shared sensorycues.

Previous hyperscanning research suggested that IBS during joint temporal planning without sensory cues is of functional relevance for successful interaction. For example, Mu et al. (2016, 2017) showed that participants pushed a button more synchronously after a jointly estimated (silent) time interval, when they had shown high IBS of centro-posterior alpha or broadly distributed gamma activity during that interval. Similarly, the pianists in our study pressed the first key after the pause more synchronously and displayed more similar tempi in the second phrase when they had exhibited higher posterior gamma IBS during the pause. In fact, gamma IBS significantly predicted whether partners’ tempi matched after the pause (while prediction of keystroke asynchronies fell short of significance). Although our data cannot provide evidence for causal links between interbrain and behavioral synchrony (Novembre and Iannetti 2021), they are in accordance with results of a recent study using dual transcranial alternating current stimulation (tACS; Novembre et al. 2017). This previous study showed that in-phase (compared to anti-phase) stimulation of the hand motor area in the beta band boosted behavioral synchrony in a joint tapping task, particularly in the initial taps directly following a silent planning period. This finding strongly suggests that IBS during joint planning constitutes a mechanism for, not a consequence of behavioral synchrony (entailing shared sensory input). Our data support this view by showing that IBS can be enhanced endogenously, without sensory cues or overt movements, through the interaction-specific alignment of internal timekeepers and temporal plans, and that this enhancement is followed at average level by more synchronous behavior.

### IBS during Joint Performance Onset

Finally, we observed a broadly distributed increase of IBS in the delta and theta band at the beginning of the first phrase, when partners had received opposite tempo instructions and were slightly less behaviorally synchronized (although IBS modulations did not predict keystroke asynchronies). As discussed above, these subtle asynchronies must have been evoked by the tempo cue at trial onset that induced anticipatory biases in internal timekeepers. Any other sensory information (e.g., the metronome or the scores) prior to performance onset was identical across conditions. However, other than in silent pauses, the incongruent tempo instruction led to an increase of IBS when pianists could hear each other.

We attribute the observed delta/theta IBS increase not to timekeeping per se, but to the compensatory increase of attention to the partner and mutual adaptive behavior upon detection of the subtle temporal mismatches between self- and other-produced sounds. Mid-frontal modulations of delta/theta power in single brains have been associated with error detection (Trujillo and Allen 2007), violated temporal predictions (Cravo et al. 2011), unexpected behavior of an interaction partner (Moreau et al. 2020), sensorimotor integration (Bland and Oddie 2001), and behavioral adaptation following errors (Cavanagh et al. 2010). A recent study associated increased theta coherence with temporal error detection, and changes in delta phase with the subsequent behavioral adaptation of movement timing (Barne et al. 2017). It seems plausible to assume that these processes were triggered by the subtle behavioral asynchronies at the onset of trials with incongruent tempo instructions. Moreover, previous dual-EEG studies in duetting guitarists reported a general increase of delta/theta IBS around performance onsets compared to later points in the performance (Lindenberger et al. 2009; Sänger et al. 2012; Müller et al. 2013; Müller and Lindenberger 2021). Joint musical entries are particularly challenging moments for coordination because interactional synchrony still needs to be established (Kawase 2014; Keller 2014; Bishop and Goebl 2015). Accordingly, participants may invest more attention and cognitive control into detecting and adapting to sensory asynchronies, possibly accounting for the IBS increase in the delta/theta range at performance onset, both in the guitarists and our pianists. The absence of IBS differences in the second half of the first phrase, when synchrony was equally tight in both conditions, lends further support for the idea that delta/theta IBS at the beginning of the phrase is related to heightened coordination demands. Future studies should explore the neural sources of delta/theta IBS to shed further light on its role in establishing behavioral synchrony at interaction onset.

### Future Directions

The present study used amplitude-based IBS as a measure that we considered suitable for tracking differences in performed and planned tempi (Fujioka et al. 2009; Nozaradan et al. 2011; Fujioka et al. 2012; Nozaradan et al. 2012; Nozaradan et al. 2015; Nozaradan et al. 2016; see Nozaradan 2014 for review; Zamm et al. 2018), as well as differences in attention to sound, that is, for capturing endogenous cognitive processes and their alignment during musical interactions. However, the exact physiological basis of amplitude-based IBS remains to be clarified, especially in the delta and theta range. While periodic amplitude fluctuations have been described in the beta and gamma range (Snyder and Large 2005; Zanto et al. 2005, 2006; Fujioka et al. 2009, 2012), our measure suggests activity modulations also in the delta and theta band, peaking on every beat or every second beat of our rhythmic task, as shown in Figure 3. It is difficult to dissociate the degree to which these modulations are driven by genuine low-frequency oscillations or evoked potentials tied to the nearly isochronous auditory/motor events of our task (i.e., all crotchets). This is indeed a subject of ongoing debate in the scientific community (e.g., Breska and Deouell 2017; Novembre and Iannetti 2018; Doelling et al. 2019). Yet, future studies might attempt to clarify this issue through ad hoc experimental manipulations or computational modeling.

Moreover, amplitude-based IBS may be less suitable for capturing correlations with some of the behavioral synchrony measures used in the present study, in particular keystroke asynchronies. Sensorimotor synchronization is achieved through two processes: phase correction (which directly reduces keystroke asynchronies without globally affecting inter-keystroke intervals) and period correction (which reduces differences in inter-keystroke intervals as well as asynchronies, see Repp and Keller 2004). It might be the case that the PLV as a measure of period matching between brain signals and calculated on amplitude envelopes fluctuating at relatively slow frequencies might better capture the dynamics of period than phase correction (Zamm et al. 2021). This could explain why we find significant correlations with IKI differences in the second phrase but not asynchrony measures. Future studies comparing amplitude-based and phase-based IBS measures that seem to relate more directly to movement asynchronies in the range of milliseconds (Dumas et al. 2010; Mu et al. 2016, 2017; Kawasaki et al. 2018) may lend further informative insights into the relationships between IBS, cognition, and behavior.

## Conclusion

This study presents evidence for endogenous, cognitive origins of IBS that go beyond the mere bottom-up locking of individual brain activity to shared sensory input. It shows that IBS can be modulated in social interactions without sensory cues during joint action planning, in musical pauses when musicians keep their internal timekeepers aligned and adjusted to partner-specific timing in order to ensure maximally synchronous behavior after the pause. Moreover, during joint performance with sound, IBS is proposed to be modulated by prioritized attending to the sounds allowing the better tracking and deeper processing of information relevant for mutual adaptation during the interaction. Taken together, the data suggest that IBS plays a functional role in establishing and maintaining behavioral synchrony and hence is likely to constitute a fundamental mechanism facilitating social interactions. The broader functional significance of this mechanism could be tested in future studies by exploring whether the current findings generalize to nonmusical interactions, such as conversation or team sports.

## Funding

Max Planck Society; Friedrich-Naumann-Stiftung für die Freiheit (to K.G.); Otto Hahn Award of the Max Planck Society (to D.S.).

## Notes

The authors wish to thank Roger Mundry, Vadim Nikulin, Mina Jamshidi Idaji, Elena Cesnaite, Tilman Stephani, Daniel Matthes, Tatsuya Daikoku, and Sven Paßmann for helpful advice on the data analysis. *Conflict of Interest*: The authors declare no competing financial interests.

## Data and Code Availability Statement

The consent form of the current study does not permit public archiving of the datasets analyzed during the experiment. Data can be made available upon request.
